# Bacteria may have multiple replication origins

**DOI:** 10.3389/fmicb.2015.00324

**Published:** 2015-04-20

**Authors:** Feng Gao

**Affiliations:** ^1^Department of Physics, Tianjin UniversityTianjin, China; ^2^Key Laboratory of Systems Bioengineering (Ministry of Education), Tianjin UniversityTianjin, China; ^3^SynBio Research Platform, Collaborative Innovation Center of Chemical Science and EngineeringTianjin, China

**Keywords:** bacteria, replication origin, Z-curve, DnaA box, DNA replication, synthetic biology, bipartite origin

## Introduction

Since the pioneer work of Woese and Fox (1977), it has been known that life on the Earth is generally classified into three main evolutionary lineages: Archaea, Bacteria, and Eukarya. In terms of DNA replication origin per chromosome, bacteria typically have a single replication origin (*oriC*), and eukaryotic organisms have multiple replication origins, whereas archaea are in between, see a recent review paper for the details (Leonard and Mechali, 2013). Among bacteria, one replication origin is the norm and there is currently no evidence that two functional origins are ever used on the same chromosome. However, it seems that there are always exceptions to the rules of biological systems. For example, Wang et al. have constructed *Escherichia coli* cells with two identical functional replication origins separated by 1 Mb in their 4.64-Mb chromosome artificially. Consequently, synchronous initiation at both spatially separate origins is followed by productive replication, and this is the first study in which cells with more than one WT origin on a bacterial chromosome have been extensively characterized (Wang et al., 2011). Recent developments in synthetic biology methodologies make the synthesis of synthetic chromosomes a feasible goal. Liang et al. fragmented the *E. coli* chromosome of 4.64 Mb into two linear autonomous replicating units with the *E. coli oriC* on the chromosome of 3.27 Mb and the replication origin of chromosome II in *Vibrio cholerae* on the chromosome of 1.37 Mb (Liang et al., 2013). Subsequently, Messerschmidt et al. also constructed the synthetic secondary *E. coli* chromosomes successfully based on the replication origin of chromosome II in *V. cholerae* (Messerschmidt et al., 2015). Recently, there are also a growing number of cases confirmed by experiments where the replication origin exists in a bipartite configuration in both Gram-positive and Gram-negative bacteria (Wolanski et al., 2015), such as Gram-positive *Bacillus subtilis* (Moriya et al., 1992) and Gram-negative *Helicobacter pylori* (Donczew et al., 2012). In addition, two autonomously replicating elements isolated from *Pseudomonas aeruginosa* have been characterized *in vitro* for pre-priming complex formation using combinations of replication proteins from *P. aeruginosa* and *E. coli* (Yee and Smith, 1990; Smith et al., 1991).

Then, could multiple replication origins occur on a bacterial chromosome? This open question has even been raised by Prof. Pavel Pevzner in a popular online course “Bioinformatics Algorithms” on Coursera (http://coursera.org/course/bioinformatics) recently. Based on the summarization of the diverse patterns of strand asymmetry among different taxonomic groups, Xia suggested that the single-origin replication may not be universal among some bacterial species that exhibit strand asymmetry patterns consistent with the multiple origins of replication (Xia, 2012). However, the strand asymmetry patterns were caused not only by replication-associated mutational pressure, and many phenomena, such as genome rearrangements, could influence the strand asymmetry patterns. Consequently, the local minima in the skew diagram do not always correspond to the positions of functional replication origins (Mackiewicz et al., 2004). Therefore, more evidences are needed to support multiple replication origins on a bacterial chromosome.

## Conserved features for typical bacterial replication origins identified by the Z-curve methodology

The Z-curve is a three-dimensional curve that constitutes a unique representation of a DNA sequence, whose components represent three independent distributions that completely describe the DNA sequence being studied. The components *x*_n_, *y*_n_, and *z*_n_, display the distributions of purine versus pyrimidine (R vs. Y), amino versus keto (M vs. K) and strong H-bond versus weak H-bond (S vs. W) bases along the DNA sequence, respectively. Among them, the *x*_n_ and *y*_n_ components are termed RY and MK disparity curves, respectively. The AT and GC disparity curves are defined by (*x*_n_ + *y*_n_)/2 and (*x*_n_ – *y*_n_)/2, which show the excess of A over T and G over C along the DNA sequence, respectively. The RY and MK disparity curves, as well as the AT and GC disparity curves, could be used to predict replication origins, since Z-curves can display the asymmetrical nucleotide distributions around *oriC*s (Zhang and Zhang, 2005; Gao, 2014). For example, the Z-curve analysis suggested the existence of multiple replication origins in archaeal genome for the first time (Zhang and Zhang, 2003), and the locations of the three predicted replication origins in *Sulfolobus solfataricus* P2 are all consistent with the results of subsequent *in vivo* studies (Lundgren et al., 2004; Robinson et al., 2004).

Based on the Z-curve method, with the means of comparative genomics, a web-based system, Ori-Finder, has been developed to identify *oriC*s in bacterial and archaeal genomes with high accuracy and reliability (Gao and Zhang, 2008; Luo et al., 2014). The predicted *oriC* regions have been organized into a database of *oriC* regions in bacterial and archaeal genomes (DoriC) (Gao and Zhang, 2007; Gao et al., 2013). Based on the predicted *oriC* regions in DoriC, conserved features for typical bacterial *oriCs* could be summarized, such as the asymmetrical nucleotide distributions around *oriC*s, the occurrence of the replication related genes adjacent to *oriC*s and the clustered DnaA boxes within *oriC*s etc. In fact, it has been noted that Ori-Finder outputs several prediction results for some bacterial chromosomes. However, only the most probable origin was presented in DoriC based on the hypothesis that bacteria only have a single replication origin, although some others also have almost all the sequence hallmarks of bacterial *oriC*s summarized above. Here, we explore the thousands of bacterial chromosomes in DoriC again, in search of multiple replication origins that comply with the above criteria on a bacterial chromosome. That is, the candidate *oriC* regions should be closely next to the replication related genes as well as the switch of Z-curves (RY, MK, AT and GC disparity curves), and contain at least three DnaA boxes. Note that only the *E. coli* perfect DnaA box (TTATCCACA) was considered with no more than one mismatch currently.

## Representative bacteria with putative double replication origins

The *oriC* information of some representative bacterial chromosomes with putative double origins of replication in DoriC is listed in Table 1. Among them, some bacteria contain double replication origins, which are located very close to each other and exhibit bipartite configuration. For example, the *oriC* regions of *Acidaminococcus fermentans* DSM 20731 are located within the *rpmH*-*dnaA*-*dnaA*-*dnaN*-*recF*-*gyrB*-*gyrA* genes cluster, next to the *dnaA* genes encoding the chromosomal replication initiator proteins. The *oriC* region is frequently within the genes cluster *rpmH*-*dnaA*-*dnaN*-*recF*-*gyrB*-*gyrA* for a great number of bacteria, usually next to the *dnaA* gene. The only difference is that two *dnaA* genes are present in the genes cluster in *A. fermentans* DSM 2073, which is a unique configuration. The two identified *oriC*s are both putative bipartite origins that are composed of two sub-regions, each of which contains a cluster of DnaA boxes (Wolanski et al., 2015). Here, the bipartite origin is split into two sub-regions by the *dnaA* gene, and 13 DnaA boxes were identified in *oriC* 1 while 20 DnaA boxes were identified in *oriC* 2. The presence of the additional *dnaA* gene and *oriC* region may be due to the chromosomal duplication, which is especially typical for *Mycobacterium bovis* BCG str. Pasteur 1173P2. Two identical copies of the *rnpA*-*rpmH*-*dnaA*-*dnaN*-*recF*-*gyrB*-*gyrA* structure have been found in its *oriC* regions.

**Table 1:** **The *oriC* information of some representative bacterial chromosomes with putative double origins of replication in DoriC**.

**Organism**	**Refseq**	**Location**	**Adjacent gene**	**No. of DnaA boxes^a^**	**Z-curves^b^**
*Acidaminococcus fermentans* DSM 20731	NC_013740	2540..2750 nt and 4140..4386 nt (*oriC* 1) 2329473..33 nt and 1195..1915 nt (*oriC* 2)	*dnaA* (2751..4139 nt; *oriC* 1) *dnaA* (34..1194 nt; *oriC* 2)	13 (*oriC* 1) 20 (*oriC* 2)	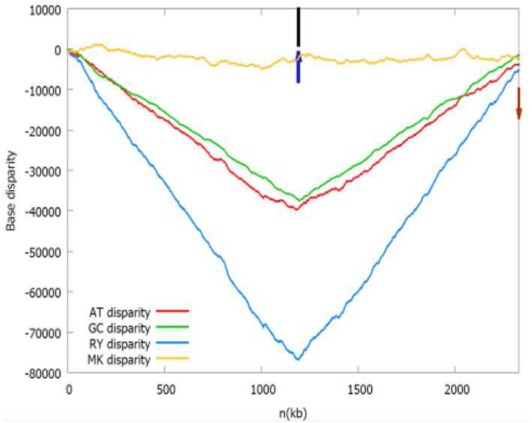
*Dehalobacter* sp. CF	NC_018867	3091418..3092048 nt (*oriC* 1) 150334..150923 nt (*oriC* 2)	*dnaA* (1..1338 nt; *oriC* 1) *parB* (149395..150333 nt; *oriC* 2)	4 (*oriC* 1) 3 (*oriC* 2)	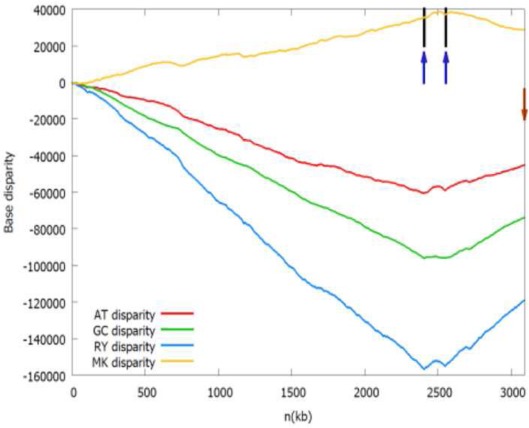
*Ralstonia pickettii* 12D chromosome 1	NC_012856	3356072..3356652 nt (*oriC* 1) 3647708..612 nt (*oriC* 2)	*hemE* (3356653..3357756 nt; *oriC* 1) *dnaA* (613..2202 nt; *oriC* 2)	3 (*oriC* 1) 3 (*oriC* 2)	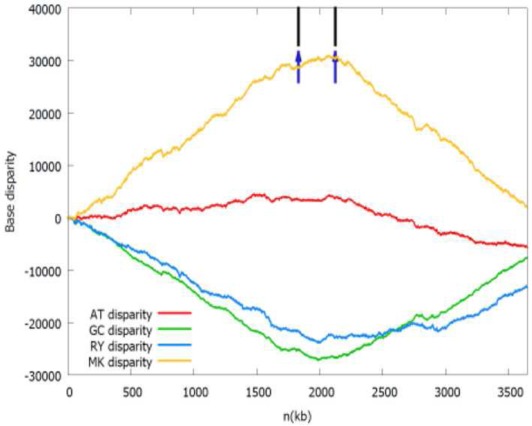
*Ochrobactrum anthropi* ATCC 49188 chromosome 1	NC_009667	544..1438 nt (*oriC* 1) 883808..884261 nt (*oriC* 2)	*dnaA* (1439..3001 nt; *oriC* 1) *hemE* (884262..885287 nt; *oriC* 2)	3 (*oriC* 1) 3 (*oriC* 2)	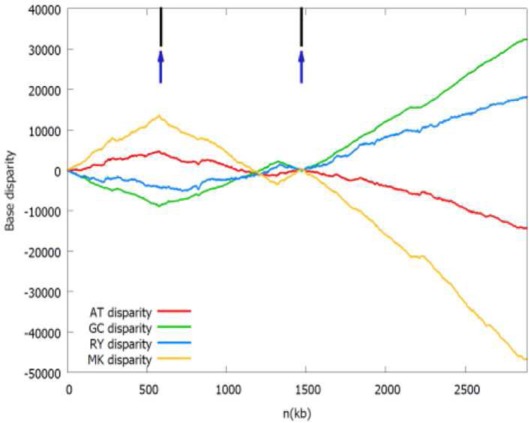

a *Note that only the E. coli perfect DnaA box (TTATCCACA) was considered with no more than one mismatch*.

b *The Z-curves (that is, RY, MK, AT, and GC disparity curves) are plotted for the rotated sequence beginning and ending in dif site or the maximum of the GC disparity curve. Short vertical black line indicates the location of the adjacent gene listed in the table, while short up vertical dark blue arrow indicates the location of the identified oriC (note that the left arrow indicates oriC 1 and the right arrow indicates oriC 2) and short down vertical brown arrow indicates dif site location, if any. It should be noted that both the black lines and dark blue arrows in the first panel (Acidaminococcus fermentans) are located too close together to be drawn individually*.

We also found *Dehalobacter* sp. CF chromosome may have two origins of replication separated by 150 kb. One is adjacent to the *dnaA* gene (*oriC* 1), and the other (*oriC* 2) is adjacent to the *parB* gene, which encodes the chromosome (plasmid) partitioning protein ParB. The *oriC* 2 is located within a putative genomic island carrying many horizontally transferred genes, such as transposase, phage integrase. Therefore, the putative *oriC* 2 may be introduced by an extrachromosomal element. These two replication origins are both located close to the local minima of the RY disparity curve as shown in the related Z-curves in Table 1.

In addition, on the chromosome 1, *Ralstonia pickettii* 12D and *Ochrobactrum anthropi* ATCC 49188 may have two separated origins of replication, which are adjacent to the *dnaA* gene and the *hemE* gene, respectively. The later condition is similar to the well-studied *oriC* of *Caulobacter crescentus* (Marczynski and Shapiro, 2002). The two replication origins of *R. pickettii* 12D and *O. anthropi* ATCC 49188 are separated by 291 and 882 kb, respectively. For *O. anthropi* ATCC 49188, the two replication origins are both located close to the local minima of the GC disparity curve, and are significantly more separated compared to the bipartite origins in *B. subtilis* and *H. pylori* that are usually close together.

As shown in the related Z-curves, the two putative replication origins in *A. fermentans* DSM 20731, *R. picketti* 12D and *Dehalobacter* sp. CF are located close to each other, which are around the global minima of the GC disparity curve. Therefore, the asymmetry pattern of replichores in these species is similar to that in most bacteria with single replication origin, and the asymmetric composition of the strands could be reflected by the V-shape of the Z-curves, where the minimum and maximum correspond to the origin and terminus of DNA replication. However, for *O. anthropi* ATCC 49188, the two putative replication origins are far apart, which are located at different local minima of the GC disparity curve. Consequently, the Z-curves exhibit strand asymmetry patterns consistent with the multiple origins of replication in archaea (Zhang and Zhang, 2003).

The *in silico* analysis presented here shows that some bacteria, although very few, may have double origins of replication per bacterial chromosome. However, there is also a possibility that not both origins of replication are functional despite the finding of the evidences, such as the clustered DnaA boxes and *dnaA* gene duplications. For example, functional analysis of two autonomously replicating chromosomal replication origins from *P. aeruginosa* has shown that only one is essential for cell viability under typical laboratory growth conditions. An alternative and intriguing possibility is that the non-functional origin was once functional but no longer used as a result of structural changes (Jiang et al., 2006). This explanation may also apply to the cases presented here, especially to the *oriC*2 of *Dehalobacter* sp. CF that may be introduced by an extrachromosomal element. Anyway, the experimental confirmation of them may provide the examples of the bacteria occurring in nature with double origins of replication and determine whether both origins of replication are functional or not, which would provide new insight into the understanding of replication mechanism of bacterial genomes and contribute to the design of synthetic bacterial genome finally.

### Conflict of interest statement

The author declares that the research was conducted in the absence of any commercial or financial relationships that could be construed as a potential conflict of interest.
